# Hypomethylation in HBV integration regions aids non-invasive surveillance to hepatocellular carcinoma by low-pass genome-wide bisulfite sequencing

**DOI:** 10.1186/s12916-020-01667-x

**Published:** 2020-08-03

**Authors:** Haikun Zhang, Peiling Dong, Shicheng Guo, Chengcheng Tao, Wei Chen, Wenmin Zhao, Jiakang Wang, Ramsey Cheung, Augusto Villanueva, Jian Fan, Huiguo Ding, Steven J. Schrodi, Dake Zhang, Changqing Zeng

**Affiliations:** 1 Key Laboratory of Genomic and Precision Medicine, Beijing Institute of Genomics, Chinese Academy of Sciences, Beijing, 100101 China; 2 University of Chinese Academy of Sciences, Beijing, 100049 China; 3 Department of Hepatology, Beijing You’an Hospital Affiliated with Capital Medical University, Beijing, 100069 China; 4 Department of Medical Genetics, University of Wisconsin-Madison, Madison, WI USA; 5 Beijing Advanced Innovation Center for Biomedical Engineering, School of Biological Science and Medical Engineering, Beihang University, Beijing, 100083 China; 6 Biology Department, Stonybrook University, Stonybrook, NY USA; 7 Department of Gastroenterology and Hepatology, VA Palo Alto Health Care System and Stanford University, Palo Alto, CA USA; 8 Liver Cancer Research Program, Division of Liver Diseases, Tisch Cancer Institute, Department of Medicine, Icahn School of Medicine at Mount Sinai, New York, NY USA; 9 Computation and Informatics in Biology and Medicine, University of Wisconsin-Madison, Madison, WI USA

**Keywords:** Cell-free DNA, Hepatocellular carcinoma, HBV integration, Low-pass WGBS, DNA methylation

## Abstract

**Background:**

Circulating cell-free DNA (cfDNA) methylation has been demonstrated to be a promising approach for non-invasive cancer diagnosis. However, the high cost of whole genome bisulfite sequencing (WGBS) hinders the clinical implementation of a methylation-based cfDNA early detection biomarker. We proposed a novel strategy in low-pass WGBS (~ 5 million reads) to detect methylation changes in circulating cell-free DNA (cfDNA) from patients with liver diseases and hepatocellular carcinoma (HCC).

**Methods:**

The effective small sequencing depth were determined by 5 pilot cfDNA samples with relative high-depth WGBS. CfDNA of 51 patients with hepatitis, cirrhosis, and HCC were conducted using low-pass WGBS. The strategy was validated in an independent WGBS cohort of 32 healthy individuals and 26 early-stage HCC patients. Fifteen paired tumor tissue and buffy coat samples were used to characterize the methylation of hepatitis B virus (HBV) integration regions and genome distribution of cfDNA.

**Results:**

A significant enrichment of cfDNA in intergenic and repeat regions, especially in previously reported HBV integration sites were observed, as a feature of cfDNA and the bias of cfDNA release. Methylation profiles nearby HBV integration sites were a better indicator for hypomethylation of tumor genome comparing to Alu and LINE (long interspersed nuclear element) repeats, and were able to facilitate the cfDNA-based HCC prediction. Hypomethylation nearby HBV integration sites (5 kb flanking) was detected in HCC patients, but not in patients with hepatitis and cirrhosis (Methyl_HBV5k_, median:0.61 vs 0.72, *P* = 0.0003). Methylation levels of integration sites certain candidate regions exhibited an area under the receiver operation curve (AUC) value > 0.85 to discriminate HCC from non-HCC samples. The validation cohort achieved the prediction performance with an AUC of 0.954.

**Conclusions:**

Hypomethylation around viral integration sites aids low-pass cfDNA WGBS to serve as a non-invasive approach for early HCC detection, and inspire future efforts on tumor surveillance for oncovirus with integration activity.

## Background

Liver cancer is the fourth cause of cancer-related mortality worldwide. In the USA, liver cancer death rate increased 43% from 7.2 to 10.3 per 100,000 between 2000 and 2016 [1, 2]. Hepatocellular carcinoma (HCC), the most frequent form of primary liver cancer, generally develops in patients with chronic liver disease due to hepatitis B virus (HBV), hepatitis C virus (HCV), alcohol abuse, or non-alcoholic fatty liver disease [3, 4]. Chronic inflammation, fibrosis, and aberrant hepatocyte regeneration favor a series of genetic and epigenetic events that culminate in multistep hepatocyte malignant transformation, through dysplastic nodules and ultimately HCC [5–7]. The high risk of HCC development in patients with cirrhosis (i.e., 2–7% annual risk) justifies the recommendation of biannual HCC surveillance with abdominal ultrasound (US) with or without serum alpha-fetoprotein (AFP) in patients at high risk [8]. Non-randomized studies suggest that early HCC detection increases the odds to receive a curative treatment and increase survival. However, the sensitivity of US and AFP is 63% to detect early-stage HCC [9], which underscores the need for improved early detection tools.

Circulating cell-free DNA (cfDNA) are small double-stranded DNA fragments [10] found in plasma, urine, saliva, cerebrospinal fluid (CSF), and other body fluids [11] originating of cell apoptosis and necrosis [12]. In many settings, analyses of cfDNA can be regarded as a way to perform a “liquid biopsy,” which have been produced promising results for genetic testing [13, 14], early cancer detection [15, 16], and prognosis prediction [17, 18]. Apoptotic and necrotic tumor cells release cfDNA into the peripheral blood, which carries tumor-related genetic and epigenetic features, including cfDNA fragment size (cfDNA_size_) [19], mutations, copy number aberrations (CNV), and methylation changes [17]. Meanwhile, cfDNA also carries tissue-specific information which provides promising abilities for tissue-of-origin mapping [19–23]. As such, cfDNA could be used as an important biomarker in clinical settings. There are different technologies to investigate methylation changes in cfDNA, including scRRBS [20] and cfMeDIPseq [22]. A number of studies have focused on cfDNA as the source of early detection biomarkers in HCC [24–29], while multiple studies have focused on cfDNA methylation in cancer diagnosis in the areas of specific biomarkers [25, 29], hypomethylation [24], and tissue of origin [26–28]. Single cytosine measurement and high accuracy have enabled whole genome bisulfite sequencing (WGBS) to become the gold standard in DNA methylation analysis [30]. One challenge in detecting cell-free circulating DNA (cfDNA) in plasma is the minor fraction of cfDNA amidst the background of total circulating DNA. This is particularly true in patients with early-stage cancers and in the minimal residual disease setting, which benefits from deep sequencing producing a more sensitive indicator for early cancer detection and surveillance [26, 28, 31]. That said, low-depth sequencing in high sample sizes is a cost-effective strategy for cohort studies [32]. Utilizing reduced sequencing depth (low-pass sequencing) and correspondingly decreased sequencing cost will be crucial to facilitate an easier clinical deployment of DNA methylation-based surveillance tools. Meanwhile, the epigenetic patterns of HBV integration regions, one of the most important features of HCC, have never been investigated in cfDNA-based diagnosis system.

In this study, we investigated of cfDNA methylation profiling at low-pass WGBS and the performance of HCC prediction. We systemically collected the most comprehensive HBV integration sites (*N* = 6072) and explored the DNA methylation state around HBV integration regions of HCC patients. We evaluated the minimum sequencing depth for long-range average methylation around collected HBV integration sites and provided the landscapes of low-pass WGBS in the liver samples from healthy individuals, hepatitis, cirrhosis, and HCC patients. Finally, we proposed DNA methylation around HBV integration regions carry utility to predict HCC from non-HCC samples.

## Methods

### Sample collection

All the blood samples of patients were collected from Beijing You’an Hospital. Healthy individuals enrolled by Beijing Institute of Genomics were collected as controls. The diagnosis of chronic hepatitis B was made according to the guidelines for the prevention and treatment of chronic hepatitis B: a 2015 update [33]. We collected age, gender, HBV status, tumor size and alanine aminotransferase (ALT) test, aspartate aminotransferase (AST) test, bilirubin test, alpha-fetoprotein (AFP) test, and other related clinical information for related samples. Meanwhile, HCC patients were classified as early and late stage according to the Barcelona Clinic Liver Cancer staging system, considering A as early stage, C and D as late stage.

### Cell-free DNA extraction

Ten microliters (ml) of whole blood was collected from each patient in Streck Cell-Free DNA BCT® tubes (Streck, Omaha, NE) and immediately shipped to Beijing Institute of Genomics. Upon arrival, the blood was collected in Streck BCT tubes which were centrifuged at 3000×*g* for 15 min at 4 °C within 2 h. Subsequently, the plasma was transferred into a fresh microcentrifuge tube, followed by a second centrifugation at 16,000×*g* for 10 min at room temperature. Five milliliters of resultant plasma was used for cfDNA extraction using a QIAamp Circulating Nucleic Acid Kit (Qiagen, Valencia, CA). After extraction, total DNA was quantified using a Qubit dsDNAHS Assay kit (Life Technologies, Grand Island, NY, USA). All DNA samples were stored at − 80 °C before sequencing library construction.

### Whole genome bisulfite sequencing and data processing

The TruSeq DNA Methylation Kit (Illumina Inc.) was used according to the manufacturers’ protocol. Total cfDNA (range from 0.5 to 88.7 ng) was used for sequencing library construction. Bisulfite conversion of cfDNA was performed using the EZ DNA Methylation-Gold Kit (Zymo Research) according to the instruction manual. During conversion, 0.5% methylated lambda DNA was included as a spike-in DNA control to estimate the conversion efficiency of unmodified cytosine. The sequencing libraries were then performed with paired-end sequencing (2 × 100 bp) on an Illumina HiSeq 4000 (Illumina Inc., San Diego, CA, USA).

After base calling, all paired-end fastq files were trimmed using cutadapt (v 1.8.3) [34] to removed adapter sequences and low-quality bases with parameters “-q 15 --minimum-length 36.” HG19 reference genome was downloaded from ENSEMBL. Lambda genome was also included in the reference sequence for calculating bisulfite conversion rate. Filtered paired-end bisulfite sequencing data were mapped with Bismark (v0.14.5) [35] using with default parameters. After alignment, read duplicates were removed using the deduplicate_bismark application included in the bismark software. Then the BAM files produced by Bismark were sorted using samtools (v0.1.19), and overlapping paired-end reads were clipped using ClipOverlap function of bamUtil (https://github.com/statgen/bamUtil) to prevent counting twice from the same observation. For each CpG, the methylation level was combined from both DNA strands and estimated as *m*/(*m* + *u*), where *m* was defined as the number of methylated cytosines and *u* was defined as the number of unmethylated cytosines. The number of methylated and unmethylated cytosines of 1 Mb regions was generated using R package methylKit. The average methylation level of each long-range region was calculated as the total number of cytosines divided by the number of methylated cytosines.

### cfDNA fragment size determination and distribution

Unique reads with well alignments to human genome (hg19) were applied for cfDNA fragment size evaluation. The end positions and start positions were extracted to calculate the cfDNA size and the distribution were prepared for different samples. Wilcoxon rank sum test was applied to test the association between the median of cfDNA_size_ in HCC and non-HCC samples.

### The enrichment score in each genomic region

The enrichment score is defined as follows: $\mathrm{Enrichment}\ \mathrm{Score}={\log}_2\left(\frac{\mathrm{DMC}}{E}\right),\mathrm{DMC}$ is the number of DMC sites in the genomic element, where the expected value $E=\frac{\left({N}_{\mathrm{DMC}}\right)\left({N}_{\mathrm{CpG}}\right)}{N_T}$, *N*_DMC_ is the number of DMC sites in the genome, *N*_CpG_ is the number of CpG sites in the genomic element, and *N*_*T*_ is the total number of CpG sites in the genome. DMCs inside and outside CpG islands are annotated according to CpG islands obtained from UCSC Genome Browser [36].

### Identification and annotation of the differentially methylated CpGs (DMCs) and genes (DMGs)

Differentially methylated CpGs (DMCs) were identified between HCC patient and healthy individual (D4 vs. D1). The identification of DMCs was generated using the R package methylKit [37]. The significance of the DMCs departure between two groups was calculated with at least 5-fold coverage. *P* value was adjusted for multiple testing with the method of Benjamini and Hochberg [38]. The CpG sites were considered different between case and control if the Benjamini-Hochberg-corrected *P* value ≤0.05 and the methylation level difference was ≥0.2. Each DMC was annotated for each RefSeq transcript obtained from ENSEMBL GRCh37. Promoters are defined as regions 2 kb upstream from TSS for each RefSeq transcript. RepeatMasker annotations were obtained from UCSC Genome Browser [36]. The HBV integration sites were extracted from previous reports [39–45].

### Calculation of average methylation level around HBV integration sites

Average methylation level of the CpGs within the 100 bp of the HBV integration sites (Methyl_HBV_) was determined in tissue samples. All the CpGs with depth over 1 read were extracted. The average methylation level within the 100 bp upstream or downstream of HBV integration sites (Methyl_HBV_) was included in all the CpGs with depth over 1 read. This value was calculated as the number of the total number of methylated cytosines divided by the number of total cytosines within the 100 bp of the HBV integration sites.

Long-range methylation around HBV integration sites (Methyl_HBV5K_) was defined as the average methylation level of the CpGs within the 5 kb of the reported HBV integration sites, calculating as the number of the total number of methylated cytosines divided by the number of total cytosines within the 5 kb of the HBV integration sites.

### Randomly resampling lower reads from total WGBS data

Regions within 5 kb of reported HBV integration sites were applied to measure the methylation status. Overlapping regions were merged to form a single region. A random sampling method was used to obtain low-depth WGBS for 5 pilot WGBS of cell-free DNA. In total, 1 M to 10 M read pairs (increasing by 1 M step) were randomly extracted from each WGBS data set. In each iteration, we randomly permuted genomic regions of 5 kb around the reported HBV integration sites using BEDTools shuffle [46]. The average methylation level of permuted regions of this randomly sampled low-pass reads and the average methylation level of permuted regions of total sequencing reads were calculated. The permutation was repeated 100 times and a correlation coefficient was adopted to measure the consistency between low-pass resampling reads and those based on total sequencing reads. For each sequencing depth, we repeated the random extraction 10 times to examine the variation of the correlation coefficient, and the difference (coefficient of variation, CV) among 10 values of the correlation coefficient was used to assess dispersion in the sampling process.

### Feature selection based on HBV integration regions

Random forest based feature selection to identify the potential high-performance biomarkers was applied in order to support Methyl_HBV5k_ to have consistent performance in low-pass WGBS data and to solve the minor release of cfDNA and the lower sensitivity in early-stage HCC. These regions should be long enough to be constantly detected at low-pass WGBS and could be suitable as markers of early stage HCC. For 6072 regions flanking 5 kb of HBV integration sites, regions with depth over 10 reads in all the 54 cfDNA samples were selected (3083), which were stable detected at low-pass sequencing. Then the neighbor regions were merged into one large region if their distance was less than 1 Mb. At last, 144 candidate merged regions with length larger than 1 Mb were selected and used for the feature selection procedure in healthy individuals and early-stage HCC patients. Feature selection was conducted using the R package “caret” based on a random forest algorithm using function “sbf” with parameters “sbfControl = sbfControl (functions=rfSBF, method=‘cv’, saveDetails=T).”

### Prediction analysis and receiver operating characteristics (ROC) curves

The AUCs measure the discrimination between HCC and non-HCC samples (healthy individuals, patients with chronic hepatitis and cirrhosis). AUC values calculated in our dataset were averaged AUC calculated across the fivefold cross-validation runs on the overall test dataset. The procedure is that the data including all the features were divided into five equal parts and each of them was set as the test dataset while the remaining as the training dataset. In the training stage, a logistic regression-based prediction model was used. Analysis of ROC curves was constructed using R package PredictABEL.

## Results

### DNA methylation around HBV integration sites mirrors the hypomethylation of HCC patients

**Fig. 1:**
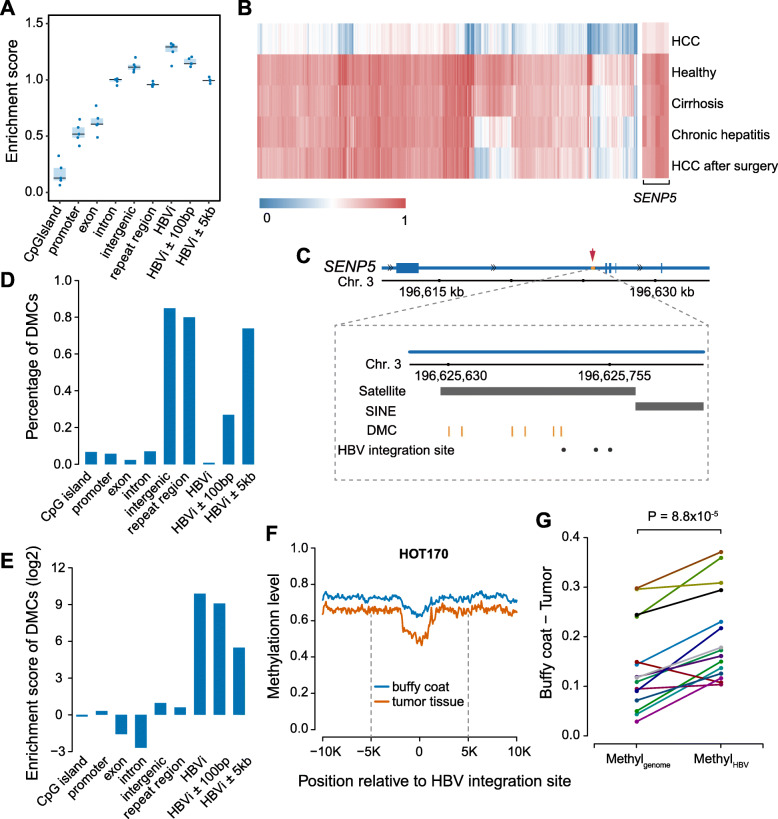
DNA methylation around HBV integration regions. **a** The enrichment score of CpGs in different genomic elements in 5 pilot WGBS. HBVi represents for HBV integration site. **b** The heatmap displays the methylation level of 2670 DMCs between HCC patient and healthy individual in all the 5 individuals. **c** The locus of 6 DMCs and 3 reported HBV integration sites in intron 2 of SENP5. The black dots represent the HBV integration sites and the orange vertical lines represent the 6 DMCs. The black bar labels represent the locus of repeat marker in this region. **d** The percentage of DMCs located at different genomic elements and regions surrounding HBV integration site. **e** The enrichment scores of DMCs at different genomic elements. **f** The average DNA methylation level profiles along 10 kb upstream and downstream of the HBV integration sites in buffy coat and tumor tissue of patient HOT170. The black dotted line represents 5 kb upstream or downstream of HBV integration sites. **g** The difference between buffy coat and tumor tissue of average methylation level across the genome (Methyl_genome_) and average methylation level of the CpGs within the 100 bp of HBV integration sites (Methyl_HBV_)

In order to explore methylation profiles in cell-free-based WGBS data, we conducted a pilot study with 5 cfDNA samples using relative high-depth WGBS: one healthy individual (D1), one patient with chronic hepatitis (D2), one patient with cirrhosis (D3), and two HCC patients (D4 and D5 of before and after surgery). The final read count equated to a mean of 58 million (M) reads per sample (Additional file 2: Table S1). The average DNA methylation across the genome (Methyl_genome_) was much lower in the HCC patient (D4; 53.56%) compared to the healthy individual and patient with chronic hepatitis and cirrhosis (74.76%, 75.13%, and 75.65%; Additional file 1: Fig. S1A; Additional file 2: Table S1). We found the genome distribution of CpGs in WGBS data tended to be located at intronic, intergenic, and repeat regions (Fig. 1a).

Next, we identified differentially methylated CpGs (DMCs) and differentially methylated gene (DMGs) with cell-free WGBS data between HCC patient and healthy individual. On average, each cfDNA sample had 7,274,674 CpGs with sequencing depth over 5 reads (Additional file 2: Table S1). In total, we identified 2670 DMCs in HCC patient compared to healthy individual (Additional file 3: Table S2), of which 99.8% were hypomethylated in HCC patients. These hypo-DMCs clearly separated the HCC patient from the healthy individual, patient with chronic hepatitis and cirrhosis, and HCC patient after surgery (Fig. 1b). Among hypo-DMCs, 174 DMCs (6.5% of 2670 DMC) were located in promoter or gene body of 77 genes. In particular, *SENP5* gene had six significantly hypomethylated DMCs with consistently high sequencing coverage across all the five individuals (an average of 295 reads; Fig. 1b and Additional file 1: Fig. S1B). Intriguingly, all six DMCs that we found in intron 2 of *SENP5* were located near previously reported HBV integration sites in HCC (Fig. 1c) [44].

We found that 80% of DMCs of HCC patients were located within repeat regions (Fig. 1d). Considering that repeat regions are a known target for HBV integration [47, 48], we analyzed the location of DMCs relative to reported HBV integration sites [39–45]. Totally, we collected 6072 HBV integration sites from published researches (Additional file 4: Table S3). Among the 2670 DMCs observed in HCC patient, 21 completely overlapped with the HBV integration sites, including one in *SENP5*. Additionally, 26.8% of the DMCs were located within a 100-bp region either upstream or downstream of integration sites, and 73.9% of DMCs were within 5 kbp (Fig. 1d). Overall, these DMCs were more enriched in HBV integration sites compared to promoter and gene coding regions (Fig. 1e, *P* < 2.2 × 10^− 16^, Fisher’s exact test). Considering the uneven distribution of CpGs inside and outside CpG islands, we calculated the enrichment score of DMCs inside and outside CpG islands, separately. Consistent with all the DMCs, both DMCs inside and outside CpG islands were more enriched in HBV integration sites (Additional file 1: Fig. S2).

Although cell-free DNA were observed to be more likely to locate at HBV integration sites (Fig. 1a, Fisher’s exact test), DMCs have higher enrichment in HBV integration sites compared to the whole cfDNA background (Fig. 1a; Fig. 1e). With above findings, we further examined whether DNA methylation levels around HBV integration regions could represent the hypomethylation of HCC genome and be used in optimization of prediction model for HCC. In HCC tumor tissues and paired buffy coat samples in a previous study [24], the hypomethalytion near the HBV integration sites were observed in both tumor and buffy coat, and the closer to integration sites, the lower methylation levels. Methylation levels were further reduced in tumor tissue, especially within the 100-bp region near these sites (Fig. 1f and Additional file 1: Fig. S3). We calculated the average methylation level of the CpGs within the 100-bp region nearby HBV integration sites (Methyl_HBV_) in each tissue sample, as the indicator for methylation level (“Methods”). Although Methyl_HBV_ was lower than the average methylation level across the genome (Methyl_genome_) in both buffy coat and tumor tissue, tumor tissue samples had a significantly smaller Methyl_HBV_ compared with buffy coat (*P* = 8.8 × 10^− 5^, *t* test). Particularly, Methyl_HBV_ was significantly lower than Methyl_genome_ in tumor tissue samples (*P* = 8.8 × 10^− 5^, t test; Fig. 1g), which supports DNA methylation around HBV integration sites as a more sensitive indicator to detect HCC compared to average methylation level across the genome.

Considering the hypomethylation of HBV integration regions in tumor tissue may be likely driven by the repeat regions well known to be hypomethylation in HCC tumors, we explored whether the methylation status of repeat elements explained the hypomethylation of HBV integrated regions. The annotations of repeat regions in HBV integration sites showed that the most overlapped repeat element is Alu and LINE (12.5% and 12.3%; Additional file 4: Table S3), and then we calculated the average methylation level of the CpGs within Alu (Methyl_Alu_), LINE (Methyl_LINE_) in paired tissue samples and compared with Methyl_HBV_. As shown in Fig. S4, Methyl_HBV_ was lower than Methyl_Alu_ in tumor tissue samples (*P* = 0.0003, t test, Additional file 1: Fig. S4). Although the average Methyl_HBV_ and Methyl_LINE_ were similar (*P* = 0.609, *t* test, Additional file 1: Fig. S4), values of Methyl_LINE_ were not constantly low across all samples, some of which had Methyl_LINE_ much higher than Methyl_HBV_. These suggested hypomethylation of HBV integration regions is not likely to be driven by surrounding repeat elements.

### Hypomethylation of regions near HBV integration sites effectively detected by a low-pass sequencing strategy in cell-free WGBS data

**Fig. 2:**
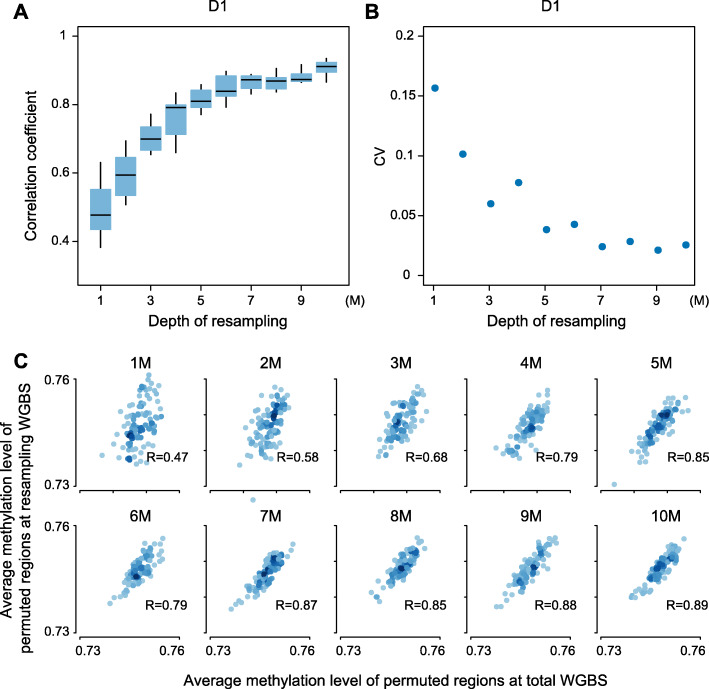
Efficacy of a low-pass sequencing strategy illustrated by resampling reads in healthy individual. **a** The boxplot shows the correlation coefficient between resampling low-pass WGBS and total sequencing reads for 10 times from 1 M to 10 M. **b** The coefficient of variation (CV) for 10 correlation coefficient between resampling low-pass WGBS and total sequencing reads from 1 M to 10 M. **c** The correlation between average methylation level of permutated regions at resampling reads and average methylation level of permutated regions at total sequencing reads from 1 M to 10 M for one time

Considering the dispersive and limited genomic regions represented by cfDNA fragments, particularly in patients with early-stage HCC, long-range methylation around HBV integration sites (Methyl_HBV5K_) was applied to measure the methylation status of cfDNA in the five cfDNA samples at high-depth sequencing volume (each composed of approximately 58 M reads). As expected, Methyl_HBV5K_ was much lower in the HCC patient (49.85%) compared to the healthy individual and patient with chronic hepatitis and cirrhosis (72.72%, 71.58%, and 71.92%; Additional file 2: Table S1; Additional file 1: Fig. S1A). To determine the effective small sequencing depth, we randomly sampled 1 M to 10 M mappable reads from each sequencing dataset and calculated permuted Methyl_HBV5K_ respectively (“Methods”). As predicted, when we used more sequencing reads, permuted Methyl_HBV5K_ was closer to the value calculated using total sequencing reads. The correlation coefficient between the methylation level from low-pass WGBS and total WGBS data saturates when using 5 M or more reads (Fig. 2a; Additional file 1: Fig. S5). The correlation coefficient at permuted regions between 5 M resampling reads and all sequencing reads was above 0.77 (Pearson’s correlation coefficient, Fig. 2, Additional file 1: Fig. S5), and the methylation level remained consistent after resampling 10 times (CV is 3.8%, 4.5%, 2.4%, 3.0%, 5.1% for D1, D2, D3, D4, and D5, respectively, Additional file 1: Fig. S5). In summary, we demonstrate that 5 M mappable reads without redundancy in low-pass WGBS is a reliable approach to evaluate the methylation level of cfDNA samples in the long-range mode.

**Fig. 3:**
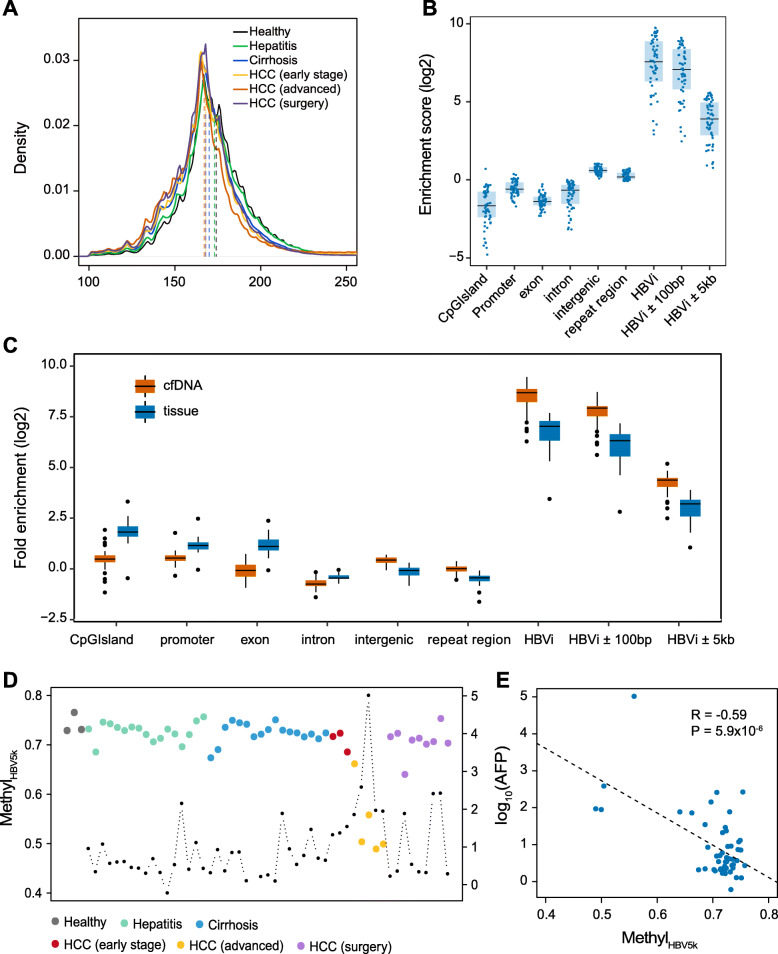
Landscape of plasma cfDNA in healthy individuals and hepatitis, cirrhosis, and HCC patients. **a** The distribution of cfDNA fragment size in the group of healthy, hepatitis, cirrhosis, early-stage HCC, advanced HCC, and HCC after surgery. The vertical dashed lines indicate the median values in all groups. **b** The enrichment scores of CpGs at different genomic elements and regions surrounding HBV integration sites of all the 54 cfDNA samples at low-pass WGBS. HBVi represents for HBV integration site. **c** The enrichment scores of CpGs at different genomic elements of cfDNA and tissue samples by randomly resampling 10 M reads from published dataset. *P* values between cfDNA samples and tissue samples at CpG island, promoter, exon, intron, intergenic, repeat region, HBV integration site, HBVi ±100 bp, and HBVi ±5 kb are 4.1 × 10^−12^, 7.6 × 10^−12^, 1.5 × 10^−13^, 4.9 × 10^−8^, 4.7 × 10^−13^, 2.1 × 10^−12^, 1.3 × 10^−11^, 9.2 × 10^−12^, and 1.9 × 10^−11^, respectively. **d** Long-range methylation around HBV integration sites (Methyl_HBV5k_) in all the 54 samples. The black dot represents for AFP level (log_10_) for the corresponding individual. **e** The correlation between AFP (log_10_) and Methyl_HBV5k_

We next sought to evaluate the ability of low-pass WGBS of cfDNA to discriminate the patients with different liver diseases. We conducted low-pass WGBS to the circulating cfDNA which are from 54 individuals, including 17 HCC (3 early-stage HCC, 5 advanced HCC, and 9 HCC patients after surgery; 16 were HBsAg positive and 1 was anti-HBs positive), 17 with cirrhosis (14 from HBV, 1 from NASH, 1 from alcohol, and 1 cryptogenic cirrhosis), 17 with hepatitis B, and 3 healthy volunteers (Additional file 5: Table S4). On average, 10.2 M mappable reads were obtained (IQR = 6.3 M, Additional file 6: Table S5). The cfDNA fragment size (cfDNA_size_) in HCC samples were significantly shorter than non-HCC samples (*P* = 0.003, Wilcoxon rank sum test), consistent with recent observation [19]. Particularly, cfDNA_size_ in advanced HCC group were much shorter than those in healthy individuals (*P* < 2.2 × 10^− 16^, Wilcoxon rank sum test; Fig. 3a), and the size seemed to decrease along with liver disease progression (Fig. 3a). As expected, the distribution of CpGs captured by low-pass WGBS also tended to be located at intergenic and repeat regions. Moreover, CpGs in low-pass WGBS had much higher enrichment score of regions around reported HBV integration sites than high-depth WGBS datasets (Fig. 3b, Fig. 1a). To figure out the enrichment at repeat regions is a feature of cfDNA or artifacts of WGBS, we randomly extracted 10 M single reads from published high-depth cfDNA WGBS datasets [24], including 58 cfDNA samples and 30 tissue samples as well as analyzed region enrichment score. Overrepresentation of regions around reported HBV integration sites was also observed in these datasets (Fig. 3c; Additional file 1: Fig. S6). Strikingly, compared to tumor tissue and buffy coat, cfDNA samples were less enriched in functional elements (CpG island, promoter, and exon) and more enriched in intergenic, repeat regions and HBV integration regions in both randomly 10 M reads and high-depth data (randomly 10 M reads in Fig. 3c; high-depth reads in Additional file 1: Fig. S6), suggesting this enrichment is a feature of cfDNA and the bias of cfDNA release.

Using our low-pass WGBS datasets, we explored whether DNA methylation in HBV integration regions could mirror the hypomethylation profiles of cfDNA from HCC patients and the potential for early HCC detection. According to Methyl_HBV5K_, the advanced HCC patients showed significantly hypomethylation level compared to healthy individuals (< 66.1%; *P* = 0.03, Wilcoxon rank sum test; Fig. 3d; Additional file 6: Table S5). However, for early-stage HCC patients, this methylation level was relatively higher, ranging from 68.5 to 72.3%. As expected, after surgery, most HCC patients (8/9) demonstrated similar cfDNA methylation levels to healthy individuals and patients with chronic hepatitis or cirrhosis. Nevertheless, one (P45) out of the nine HCC patients exhibited a lower methylation after surgery (63.97%, Fig. 3d; Additional file 6: Table S5) and died 2 months later due to tumor recurrence, suggesting that there were micro-metastasis with tumor cells in that individual. Additionally, a negative correlation was observed between Methyl_HBV5K_ and alpha-fetoprotein (AFP) levels (Pearson’s correlation coefficient = − 0.59, *P* = 5.9 × 10^− 6^; Fig. 3d, e). Besides, Methyl_HBV5K_ seemed to have no difference among healthy individuals and patients with chronic hepatitis and cirrhosis (*P* > 0.1, Wilcoxon rank sum test). We also included one patient with acute hepatitis B in the hepatitis group and found that Methyl_HBV5K_ from this patient was similar to patients with chronic hepatitis (Fig. 3d; Additional file 6: Table S5).

### DNA methylation around HBV integration regions aid HCC prediction

**Fig. 4:**
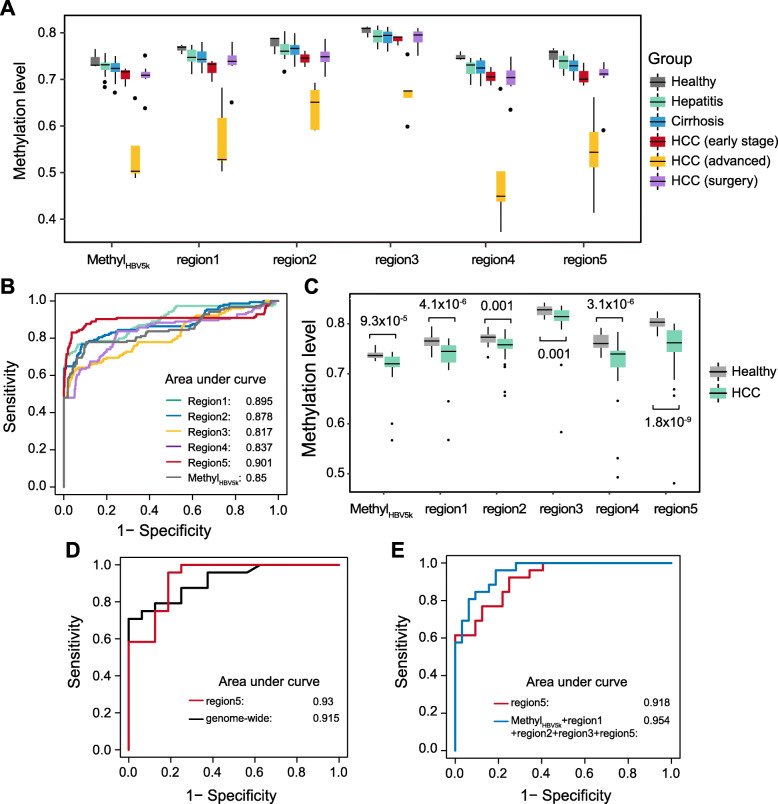
Hypomethylation around HBV integration regions aid HCC prediction. **a** Boxplot displays Methyl_HBV5k_ and the methylation level of top 5 selected regions in all the 54 samples. **b** Receiver operating characteristics (ROC) curve based on fivefold cross-validation for HCC detection by different indicators in discriminating HCC patients from individuals without HCC (healthy individuals, patients with hepatitis and cirrhosis). **c** The comparisons between healthy individuals and patients with early-stage HCC using Methyl_HBV5k_ and the methylation level of top 5 selected regions in the validation dataset. **d** ROC curves for 16 healthy individuals and 24 HCC patients in the validation cohort using genome-wide hypomethylation analysis and region 5. **e** ROC curves for HCC patient detection using all the healthy individuals and HCC patients in the validation cohort by the identified features

We evaluated Methyl_HBV5K_ by their differentiation ability to HCC from non-HCC cfDNA samples using receiver operating characteristic (ROC) curves based on a logistic regression model by fivefold cross-validation. Methyl_HBV5K_ showed the distinguish ability of HCC from non-HCC with AUC = 0.85. We also applied random forest-based feature selection to identify the potential high-performance biomarkers (“Methods”). Top 5 regions were identified in distinguishing patients from healthy individuals (chr13: 19442162–20,713,822; chr1: 10121993–12,279,387; chr10: 11149668–13,266,296; chr10: 38027603–39,151,628; chr10: 84035111–85,772,043). All our cfDNA samples had these regions well sequenced, with the minimum amount of sequencing reads at 1991 (Additional file 7: Table S6). Their methylation levels were significantly lower in either early-stage or advanced HCC patients than in healthy individuals, and demonstrated obvious decreasing tendency along with disease progression (Fig. 4a; Additional file 7: Table S6). Further investigation showed the prediction model using regions 1, 2, and 5 could reach better performance for HCC patients (AUC > 0.85; Fig. 4b). All these prediction models exhibited improved discrimination performance compared to clinical variables (ALT, AST, Tbil, AFP) (Additional file 1: Fig. S7A).

To validate our findings, we applied this method in an independent cohort in a previous study [24]. This cfDNA cohort was comprised of 32 healthy individuals and 26 HCC patients with early stage (BCLC stage is A or B) based on single-end bisulfite sequencing. To achieve similar sequencing depth, we randomly sampled 10 M reads from each plasma sample. All the important features identified in above model showed significantly decreasing methylation in early-stage HCC compared to healthy individuals (*P* < 0.001, Wilcoxon rank sum test; Fig. 4c; Additional file 8: Table S7). The above established prediction model demonstrated competitive performance in HCC detection with genome-wide hypomethylation analysis (AUC = 0.93 and 0.91, *P* = 0.734, DeLong test; Fig. 4d). With all the 58 cfDNA samples, region 5 was still the best HCC indicator (AUC = 0.918, Additional file 1: Fig. S7B). Moreover, the combination of multiple features provided improved prediction for HCC. When combing Methyl_HBV5K_, region 1, region 2, region 3, and region 5 all together in the prediction model, it provided the best prediction performance with AUC = 0.954 (Fig. 4e).

## Discussion

In summary, we found cfDNA prefer enriched in intergenic, repeat regions and previously reported HBV integration regions indicating a non-random feature of cfDNA releasing from solid tissues. Furthermore, we demonstrated a long range of DNA methylation around HBV integration regions was a sensitive indicator to detect HCC compared to average methylation level across the genome. Hypomethylation of these regions are independent of integration events, which make them either suitable for the occurrence of viral integration, or ensure the transcription activity of integration sites recently attracting a lot of interests [49]. We demonstrate that DNA methylation around HBV integration regions could serve as HCC detection biomarkers. We also demonstrated DNA methylation around HBV integration regions reflected genome-wide demethylation changes from non-tumoral tissues to HCC and could be used as a low-cost approach detecting minimal tumoral residual disease after surgical resection. In summary, our study provided a novel low-cost HCC diagnosis strategy in which HBV integration regions were employed, and this strategy will also be promising for similar attempts in a lot of oncovirus also known to have integration ability during infection [50].

Patients with chronic liver disease are at risk of HCC development, highest among those with cirrhosis. Professional societies recommend HCC surveillance in those patients at high risk who will benefit from early diagnosis so they might receive curative therapies. The recommended strategy for surveillance includes abdominal ultrasound with or without alpha-fetoprotein (AFP) every 6 months. However, image examination required special equipment (the ultrasound machine) and trained personnel to perform and interpret the study, potential barriers especially considering the large population of patients with HBV infection in China. Ultrasound is also operator dependent. Therefore, there is an unmet clinical need for new non-invasive diagnostic tests that is not operator dependent, such as liquid biopsy using circulating tumor cells [51]. Unfortunately, The European Association for the Study of the Liver did not recommend the use of any existing tumor markers such as AFP and L3 fraction for HCC surveillance due to their suboptimal performance for early detection, and in the prior version of the American Association for the Liver Diseases, AFP was felt to lack both sensitivity or specificity for early detection of HCC. Subjects at highest risk for HCC are those with chronic hepatitis and advanced fibrosis; hepatic inflammation can result in elevation of AFP and up to 30% of HCC was non-AFP producing. Current study found a strong negative correlation between Methyl_HBV5k_ and AFP levels. However, unlike AFP, the Methyl_HBV5k_ level was not affected by the presence of inflammation, hence making it a more specific tumor marker. Currently new blood-based measurements are commonly compared with AFP, which had already been shown to have inadequate sensitivity and specificity, hence we believe future comparison should be between new biomarkers and ultrasound for early detection of HCC. Although WGBS of cfDNA has been shown effective for cancer detection [27], the cost of cfDNA WGBS in cancer patients is one of the challenges for wide application. In this paper, we explored the cfDNA methylome of hepatitis, cirrhosis, and HCC patients and examined the feasibility of HCC detection using low-pass WGBS. We demonstrated the measurement of DNA methylation around HBV integration regions could be applied in low-pass cell-free WGBS at 5 million reads to reflect liver disease status of chronic hepatitis, cirrhosis, and HCC. Moreover, DNA hypomethylation in HBV integration regions has shown promising results as a potential biomarker for early HCC detection.

Previous studies have been shown that the fragmentation process of cfDNA is not random [52, 53]. Our results show low-pass WGBS for cfDNA tended to capture fragments from repeat regions and HBV integration sites. Because open chromatin regions are easily degraded, fragments from open chromatin regions (promoter and gene coding regions) were less likely to be detected in cfDNA. When decreasing the sequencing volume, overrepresentation of genomic repeat regions and HBV integration regions was observed in cfDNA. This suggests that the signal from these regions could remain given adequate sequencing depth in low-pass WGBS. Since HBV integrations tend to localize at repeat regions, DMCs of advanced HCC patient were also enriched in previously reported HBV integration sites.

We adopted an approach focusing on regions from HBV integration sites as surrogate regions for plasma hypomethylation analysis in HCC patients. Although we chose HBV integration sites as the indicator, it does not necessarily indicate that the analysis is only suitable for patients with HBV infection. In our sample set, we also included three patients without HBV infection (P1, P18, and P19; Additional file 5: Table S4). While HBV integrations carried by dominant tumor clones are likely to have some specific DNA molecular features [25, 54–56], we also demonstrated that methylation changes in HBV integration regions may be common in HCC and independent of HBV infection. Interestingly, we found hypomethylation in HBV integration regions have higher sensitivity for HCC diagnosis. For example, one chronic hepatitis patient, P14, had the Methyl_HBV5k_ at 69.5%, the methylation level of region 5 at 72.4%, and an abnormal AFP level (141.9 ng/ml). The corresponding P14 blood sample was initially labeled as chronic hepatitis since he was a follow-up patient with chronic HBV infection; however, he was diagnosed with HCC in this examination and died 8 months later. Therefore, it is plausible that the patient had significant circulating tumor cells at the time of sample collection since his AFP was also significantly elevated. Except P14, the sample from a chronic hepatitis patient, P2, showed that the methylation level of region 5 was 70.7% and the Methyl_HBV5k_ was 68.5%. Using the sample from a clinical visit 6 months following the initial sample collection, the methylation level of region 5 increased to 73.92%, whereas the Methyl_HBV5k_ increased to 71.34%. This patient had no detected HCC in follow-up. As a predictor of HCC, the most challenging aspect is to determine appropriate cutoffs for disease status, which necessitates large sample sizes in future studies. Nevertheless, our study successfully illustrated that it is necessary to monitor the patients with suspicious methylation changes in cfDNA according to multiple indicators, combining their prognostic signals to improve accuracy. We compared our strategy with genome-wide hypomethylation analysis in a published dataset, and our strategy had competitive classification performance with the genome-wide hypomethylation analysis used in the original publication [24]. Moreover, the calculation of methylation in these regions does not rely on a reference panel of healthy individuals and is thus independent of either sequencing quality and inclusion criteria of the reference panel.

Target sequencing have already achieved certain progress in tumor detection, but genome-wide characterization of methylation profiles is the promising direction to overcome the false negative errors due to tumor heterogeneity and optimize the genomic regions used for surrogating the methylation level changes specific to tumor patients, such as previously reported HBV integration sites in our observation. We believe low-pass WGBS will facilitate efforts using large sample size for novel solutions and finally improve the clinical implementation of methylation evaluation. Although we have found some stable methylation patterns using low-pass WGBS using the fivefold cross-validation in the training set and testing the results in an independent cohort, the results indicate there may be some level of overfitting in the test data set, hence the generalization of our strategy should be further validated in larger studies in the future. The low-coverage caused by the low-pass WGBS sequencing introduced analysis challenges; however, it may still have clinical utility in augmenting early detection of HCC. This study can serve as a platform to motivate further development of low-pass DNA methylation approaches to improve the accuracy of HCC diagnoses and surveillance. Subsequent larger studies will aid in the determination of accurate cutoff values for disease stages, especially for those with small tumors. Furthermore, we anticipate that blood samples from HCC patients at multiple time points hold strong utility in tracking disease progression.

## Conclusions

We have proposed a novel strategy in which we utilized DNA methylation around HBV integration regions to apply low-pass WGBS to monitor DNA methylation levels in cfDNA fragments generated by liver disease and hepatocellular carcinoma. Overrepresentation of cfDNA fragments in intergenic, repeat regions, and HBV integration regions compared to functional elements (promoter and gene coding regions) provide additional insights into the mechanisms of HCC molecular pathophysiology and may aid in early HCC diagnosis and clinical decisions. HBV integration-based DNA methylation in cfDNA exhibited excellent predictive performance for detection of HCC, which shows utility as stable and powerful diagnostic biomarkers for cancer surveillance in liver diseases ranging from hepatitis, cirrhosis, and early-stage and advanced hepatocellular carcinoma. It will broaden clinical implementation of WGBS as a methylation-based cfDNA early detection biomarker for liver cancer and inspire future efforts on tumor surveillance for cancer-causing viruses.

## Supplementary information

**Additional file 1:.** Fig. S1. Methylation profiling of 5 pilot cfDNA samples with relative high-depth WGBS. Fig. S2. The percentage and enrichment score of DMCs inside and outside CpG islands at different genomic elements. Fig. S3. The average DNA methylation level profiles along 10 kb upstream and downstream of the HBV integration sites in all the tumor tissues and paired buffy coat samples. Fig. S4. The difference between buffy coat and tumor tissue of Methyl_genome_, Methyl_HBV_, Methyl_Alu_ and Methyl_LINE_. Fig. S5. The efficiency of re-sampling sequencing reads for low pass WGBS in 5 pilot cfDNA samples. Fig. S6. The enrichment scores of CpGs at different genomic elements by total sequencing reads from published dataset. Fig. S7. ROC curves for HCC detection using hypomethylation around HBV integration regions.

**Additional file 2:.** Table S1. The statistical information of 5 pilot WGBS samples.

**Additional file 3:.** Table S2. DMCs between HCC patient and healthy individual.

**Additional file 4:.** Table S3. The coordinates of the extracted HBV integration sites.

**Additional file 5:.** Table S4. Clinical information of 54 individuals by low-pass WGBS.

**Additional file 6:.** Table S5. The statistical information of 54 cfDNA samples by low-pass WGBS.

**Additional file 7.** Table S6. Methyl_HBV5k_ and top 5 selected regions of 54 cfDNA samples by low-pass WGBS.

**Additional file 8.** Table S7. Methyl_HBV5k_ and top 5 selected regions of 58 cfDNA samples from validation cohort by resampling 10 M single reads.

## Data Availability

The datasets supporting the conclusions of this article are available in the Genome Sequence Archive in BIG Data Center, Beijing Institute of Genomics (BIG), Chinese Academy of Sciences, under accession numbers CRA001537, CRA001537 that are publicly accessible at http://bigd.big.ac.cn/gsa.

## Abbreviations

HCC: Hepatocellular carcinoma
cfDNA: Circulating cell-free DNA
cfDNA_size_: Fragment size of circulating cell-free DNA
Methy_HBV5k_: Long-range methylation around HBV integration sites
DMCs: Differentially methylated CpGs
HBV: Hepatitis B virus
WGBS: Whole genome bisulfite sequencing
