# Supplementary material for: Hypomethylation in HBV integration regions aids non-invasive surveillance to hepatocellular carcinoma by low-pass genome-wide bisulfite sequencing
Source: BMC Med. 2020 Aug 3;18:200. doi: 10.1186/s12916-020-01667-x (PMC7397586; doi:10.1186/s12916-020-01667-x)
Supplement: Supplementary file 1 — Additional file 1:. Fig. S1. Methylation profiling of 5 pilot cfDNA samples with relative high-depth WGBS. Fig. S2. The percentage and enrichment score of DMCs inside and outside CpG islands at different genomic elements. Fig. S3. The average DNA methylation level profiles along 10 kb upstream and downstream of the HBV integration sites in all the tumor tissues and paired buffy coat samples. Fig. S4. The difference between buffy coat and tumor tissue of Methylgenome, MethylHBV, MethylAlu and MethylLINE. Fig. S5. The efficiency of re-sampling sequencing reads for low pass WGBS in 5 pilot cfDNA samples. Fig. S6. The enrichment scores of CpGs at different genomic elements by total sequencing reads from published dataset. Fig. S7. ROC curves for HCC detection using hypomethylation around HBV integration regions. [file 12916_2020_1667_MOESM1_ESM.pdf]

Supplemental figures

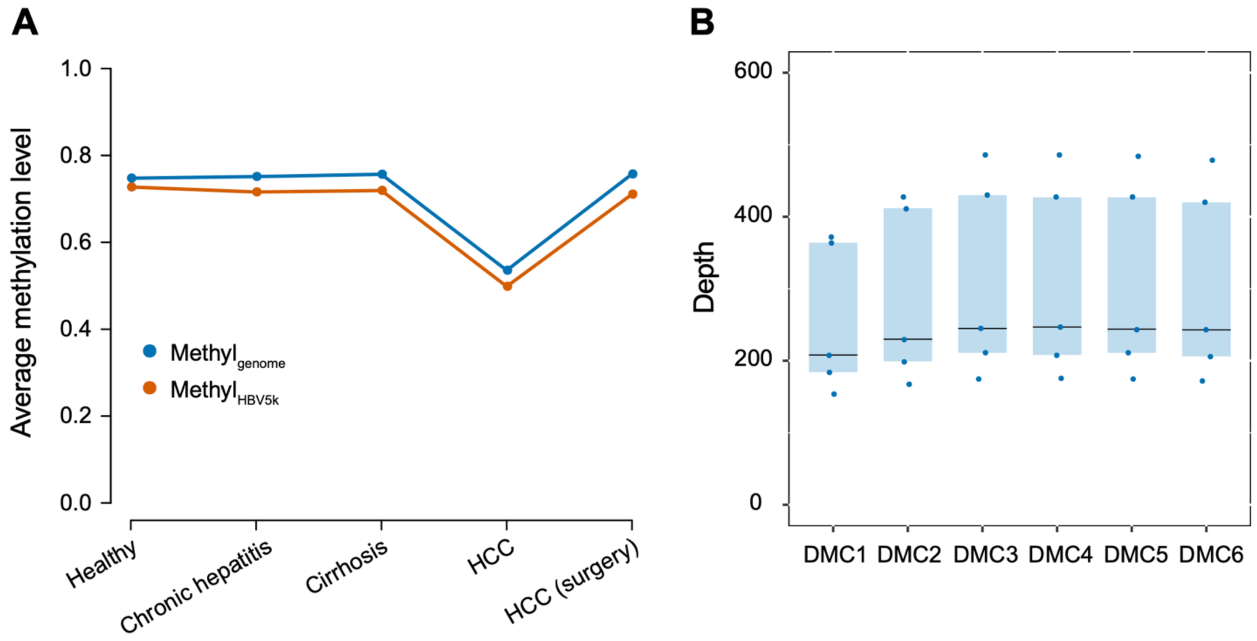

Fig. S1. Methylation profiling of 5 pilot cfDNA samples with relative high-depth WGBS. (A) The average methylation level across the genome ($Methyl_{genome}$) and long-range methylation around HBV integration sites ($Methyl_{HBV5K}$) of 5 cfDNA samples. (B) The depth of 6 DMCs of *SENP5* in 5 cfDNA samples.

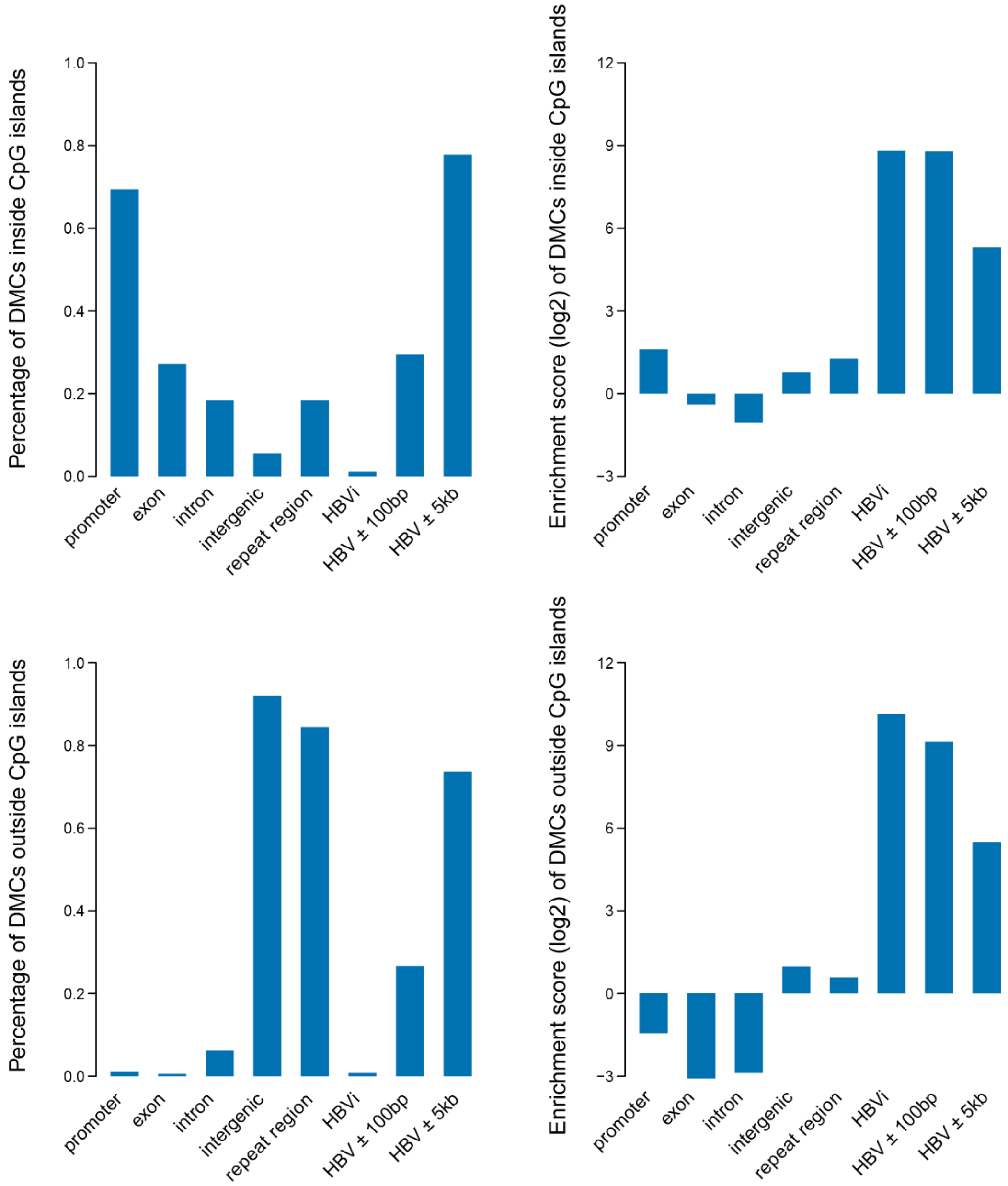

Fig. S2. The percentage and enrichment score of DMCs inside and outside CpG islands at different genomic elements.

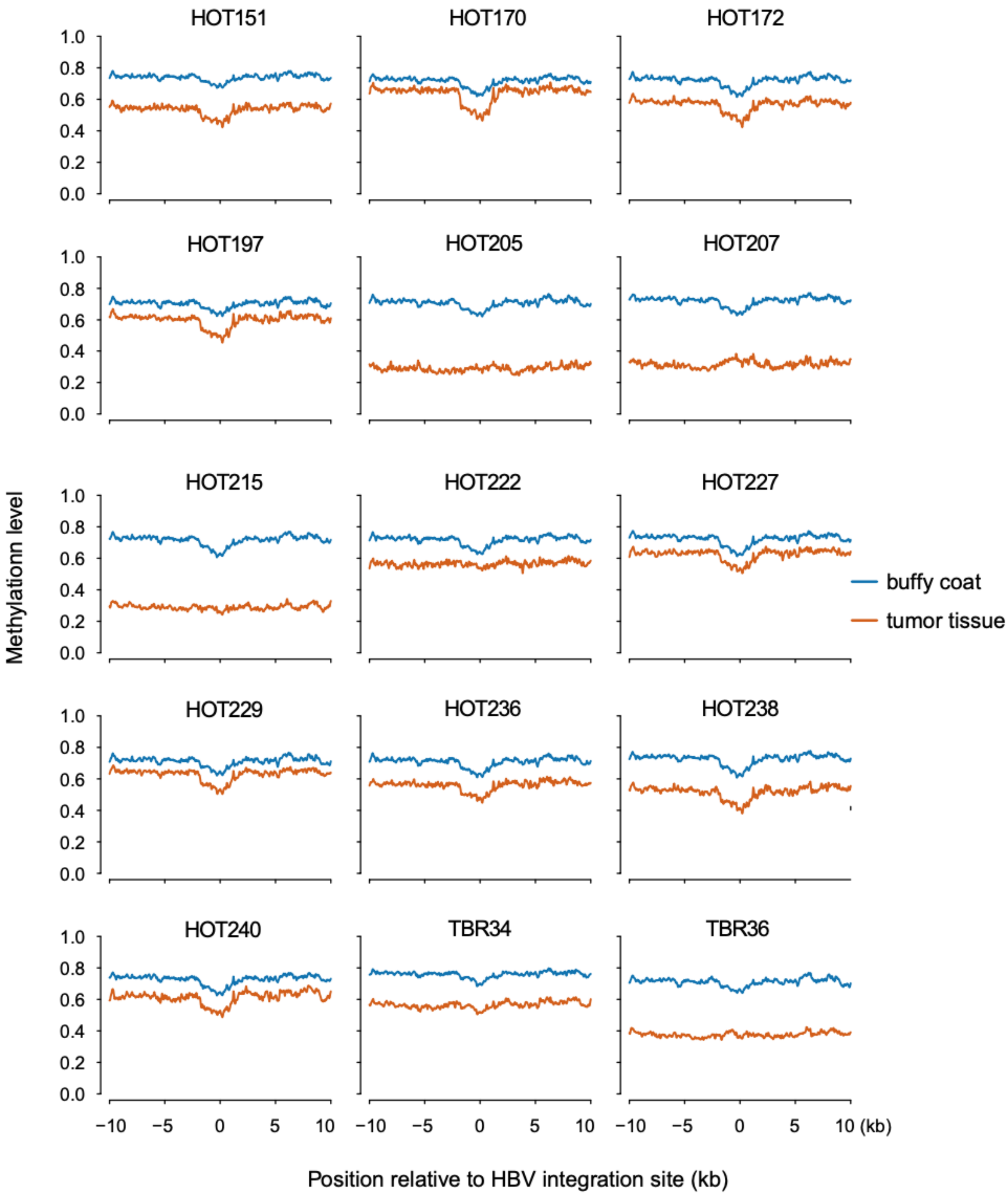

Fig. S3. The average DNA methylation level profiles along 10 kb upstream and downstream of the HBV integration sites in all the tumor tissues and paired buffy coat samples.

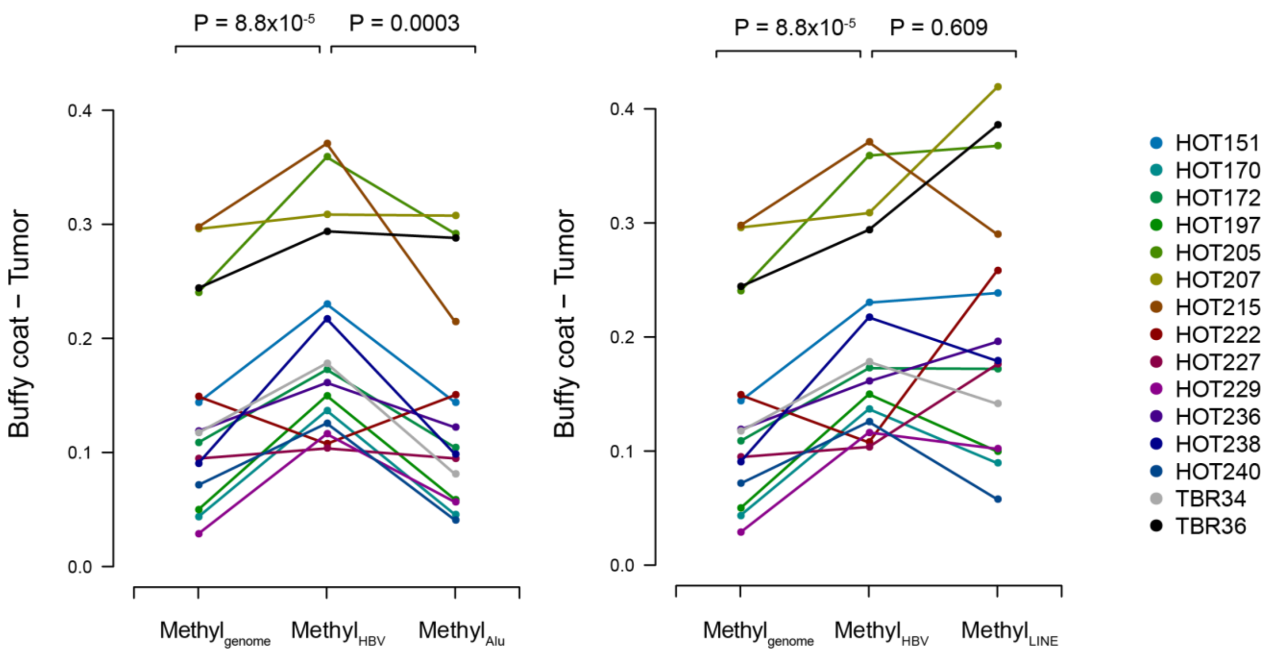

Fig. S4. The difference between buffy coat and tumor tissue of $Methyl_{genome}$, $Methyl_{HBV}$, $Methyl_{Alu}$ and $Methyl_{LINE}$.

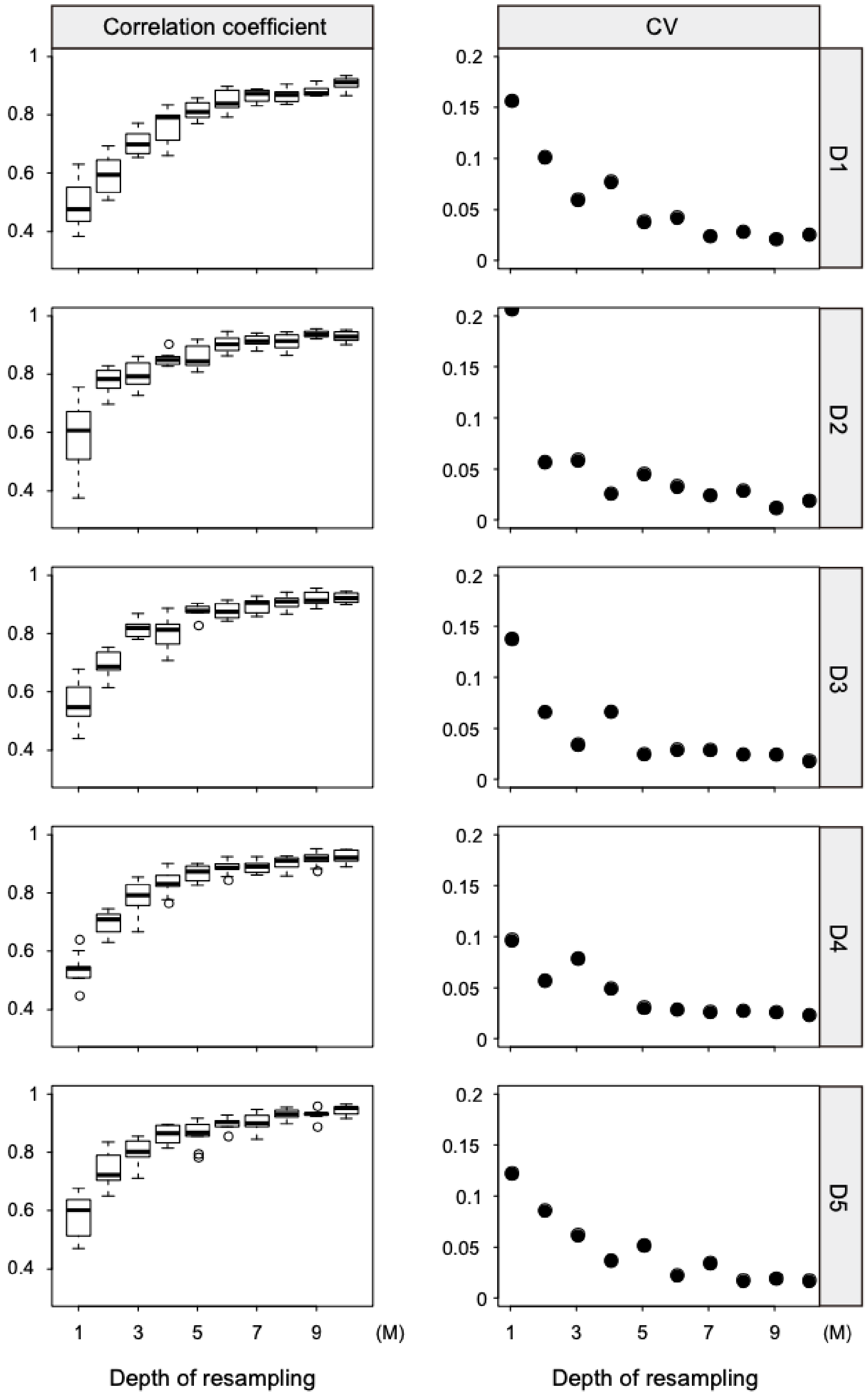

Fig. S5. The efficiency of re-sampling sequencing reads for low pass WGBS in 5 pilot cfDNA samples. Left of the figure showed the correlation coefficient between re-sampling low-pass WGBS and total sequencing reads for 10 times from 1M to 10M. Right of the figure showed the coefficient of variation (CV) for 10 correlation coefficient between re-sampling low pass WGBS and total sequencing reads from 1M to 10M.

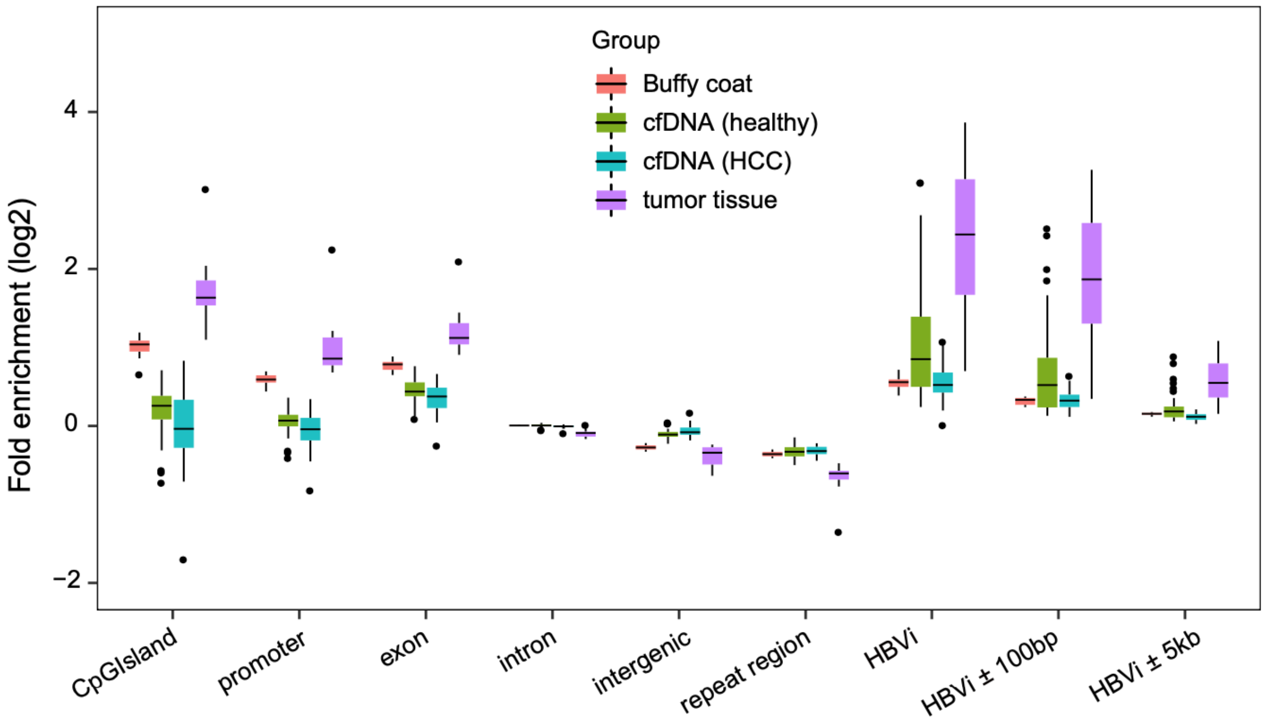

Fig. S6. The enrichment scores of CpGs at different genomic elements by total sequencing reads from published dataset. The enrichment scores of CpGs at different genomic elements of cfDNA from healthy individuals, cfDNA from early stage HCC patients, tumor tissue samples and buffy coat samples by total sequencing reads from published dataset. The high enrichment score of tumor tissue at HBV integration regions due to the lower sequencing depth in the original dataset.

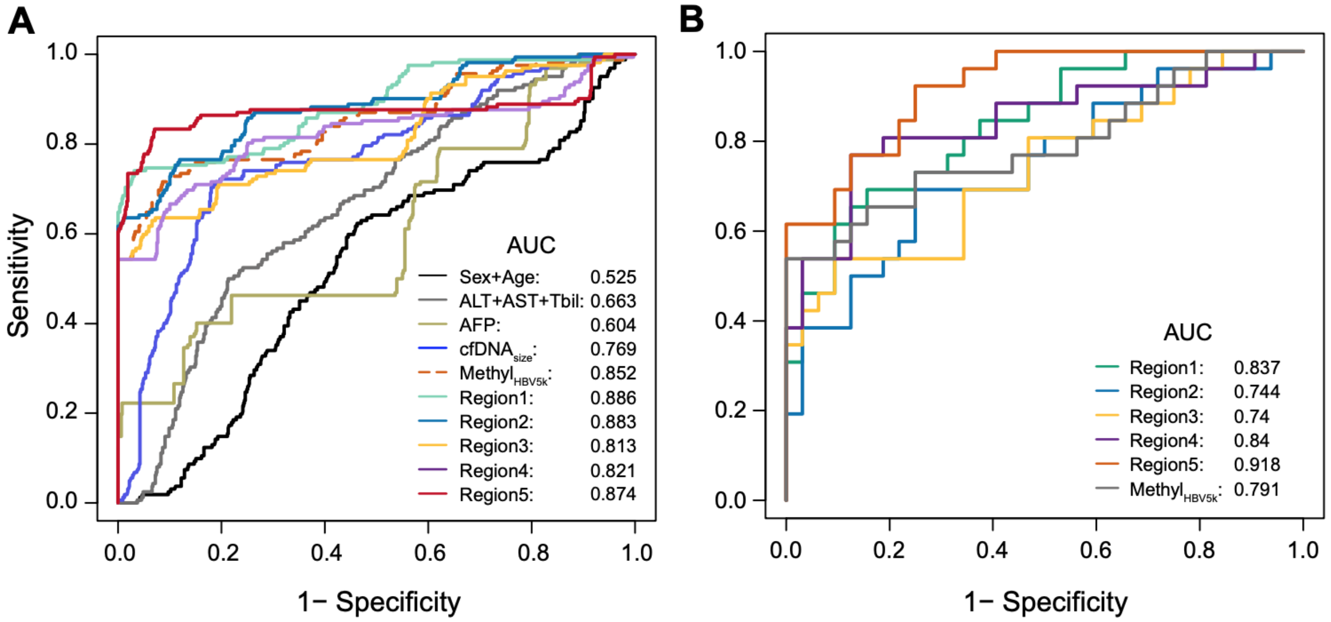

Fig. S7. ROC curves for HCC detection using hypomethylation around HBV integration regions. (A) ROC curves based on five-fold cross-validation for HCC patient detection by different indicators in discriminating HCC patients from individuals without HCC. (B) ROC curves for HCC patient detection using all the healthy individuals and HCC patients in the validation cohort by the identified features.
